# Preterm Delivery in the Setting of Left Calyceal Rupture

**DOI:** 10.1155/2015/906073

**Published:** 2015-09-21

**Authors:** Brent Hanson, Rami Tabbarah

**Affiliations:** Inova Fairfax Hospital Department of Obstetrics and Gynecology, 3300 Gallows Road, Falls Church, VA 22042, USA

## Abstract

Spontaneous rupture of the renal collecting system is a rare but serious complication of pregnancy. We report a case of nontraumatic left renal calyceal rupture in a pregnancy which ultimately progressed to preterm delivery. A 29-year-old primigravida with a remote history of urolithiasis presented with left flank pain, suprapubic pain, and signs of preterm labor at 33 weeks of gestation. The patient was believed to have urolithiasis, although initial renal ultrasound failed to demonstrate definitive calculi. After a temporary improvement in flank pain with medication, the patient experienced acute worsening of her left flank pain. Urology was consulted and further imaging was obtained. Magnetic resonance imaging (MRI) was consistent with bilateral hydronephrosis and rupture of the left renal calyx. Given the patient's worsening pain in the setting of left calyceal rupture, the urology team planned for placement of a left ureteral stent. However, before the patient could receive her stent, she progressed to active labor and delivered a viable female infant vaginally. Following delivery, the patient's flank pain resolved rapidly and spontaneously, so no surgical intervention was performed. A summary of the literature and the details of this specific clinical situation are provided.

## 1. Introduction

Spontaneous rupture of the renal collecting system during pregnancy is an uncommon occurrence. We present the 13th documented case of left-sided renal calyceal rupture in a pregnant woman. While rupture of the upper urinary tract can occur with or without an underlying cause, our patient had a history of urolithiasis and was believed to have urolithiasis during her hospitalization. In addition, pregnancy itself is known to be a risk factor for the development of obstruction of the renal system, further increasing this patient's chances for calyceal rupture. The patient failed initial conservative management and progressed to spontaneous preterm vaginal delivery before surgical stenting was performed. A review of the literature on this topic is included.

## 2. Case Presentation

A 29-year-old primigravida presented at 33 weeks of gestation in January 2015 with a four-day history of suprapubic pain and worsening intermittent left flank pain. Additionally, she endorsed headache, nausea, and vomiting during the 24 hours prior to admission. She had no fever, vaginal bleeding, leakage of fluid, diarrhea, or constipation. She endorsed good fetal movement. Her prenatal course had been uncomplicated. She endorsed a history of a urinary tract infection two years prior to her current pregnancy and a remote history of urolithiasis which was managed conservatively. On physical examination she had mild left flank tenderness but no abdominal or suprapubic tenderness. There were no peritoneal signs. Her vital signs including blood pressure were within normal limits. A urine dipstick showed moderate leukocyte esterase, 6–10 squamous cells per high power field, no blood, and no nitrites. Urine culture later documented no bacterial growth. Her white blood cell count was 12,900. Creatinine was normal. Fetal fibronectin was negative. Laboratory findings indicated normal liver and biliary function. The cardiotocogram showed rare contractions approximately every 30 minutes and a reactive fetal heart tracing. While in the labor and delivery triage area, the patient experienced an increase in the frequency of her contractions to every 5–10 minutes and underwent cervical change to greater than one centimeter in dilation, so she was admitted for management of preterm labor. Betamethasone was administered to expedite fetal lung maturity. Although this patient was beyond 32 weeks of gestation, magnesium sulfate was administered for fetal neuroprotection to decrease risk of cerebral palsy as well as for the added benefit of tocolysis.

The patient's contractions subsequently resolved while receiving magnesium, she experienced no further cervical change, and the previously reported flank pain had improved. She received her second dose of betamethasone and the magnesium was discontinued after 24 hours. On hospital day two, an ultrasound confirmed normal amniotic fluid index and fetal biophysical profile, so she was transferred from labor and delivery to the high risk perinatal unit for further observation. On hospital day three, the patient experienced acute worsening of colicky left flank pain associated with nausea and vomiting. Given the patient's negative urine culture, lack of fever, and stable white blood cell count, infectious processes such as pyelonephritis were determined to be less likely, so no antibiotics were administered. Urolithiasis was suspected, and renal ultrasound was obtained which showed mild left-sided pelviectasis not out of proportion with normal pregnancy. While no definitive stones were visualized, there were small areas of echogenicity documented in the left kidney which could represent multiple small nonshadowing stones. A repeat urinalysis showed trace blood and trace leukocyte esterase. Inpatient management was continued for analgesia and administration of intravenous fluids due to suspected urolithiasis. Gastrointestinal causes of pain were deemed less likely given the patient's location of pain in the left flank, good bowel sounds, lack of abdominal distention, and normal markers of gastrointestinal and liver function on lab work.

The patient's symptoms improved with intravenous pain medication, and she was transitioned successfully to oral pain management. On hospital day five, the patient experienced another episode of left flank pain, so urology was consulted and further imaging was obtained. A repeat renal ultrasound on hospital day five showed no significant change of the mild left hydronephrosis from previous ultrasound and no discrete abscess or stone, but MRI later the same day showed left-sided perinephric fluid and retroperitoneal fluid consistent with left calyceal rupture and urine leak. No calculus was appreciated on MRI. While awaiting the results of the MRI, the patient's symptoms improved dramatically. Vital signs were stable, her white blood cell count was trending down, and her serum creatinine was normal. In conjunction with the urology and interventional radiology teams, the options of placing a percutaneous nephrostomy or ureteral stent were discussed with the patient, but given the patient's clinical improvement, no surgical intervention was performed.

On hospital day six, the patient experienced another acute episode of flank pain, so the urology team planned to proceed with ureteral stent placement. However, before stent placement could be performed, the patient had spontaneous rupture of membranes and entered active labor. She was transferred to labor and delivery, and she had an uneventful spontaneous vaginal delivery less than 3 hours after membrane rupture. During delivery, the patient remained afebrile with no evidence of anterior abdominal tenderness. Amniotic fluid was clear and odorless, and visual inspection of the placenta after delivery showed a normal, intact placenta. Suspicion for intrauterine infection was low, so the placenta was not evaluated pathologically for evidence of chorioamnionitis. Further, the patient was documented to be GBS negative, so no antibiotics were administered during labor. Following delivery, the patient's flank pain resolved and computed tomography (CT) of the abdomen and pelvis showed only mild left perinephric stranding with a minimal amount of stranding along the left retroperitoneum. No fluid collection was seen in the retroperitoneum. No surgical intervention was performed, and the patient was discharged without symptoms on postpartum day two.

## 3. Discussion

Flank pain during pregnancy is a common complaint with a broad differential diagnosis. Common renal causes of flank pain during pregnancy include acute hydronephrosis, acute urolithiasis, and pyelonephritis [1, 2]. A broad differential diagnosis of flank pain in pregnancy must be considered, including cystitis, pyelonephritis, urethritis, nephrolithiasis, renal cysts, renal malignancy, constipation, gastrointestinal infections, appendicitis, gallbladder disease, trauma or rupture of the spleen, heterotopic or ectopic pregnancy, ovarian cysts, ovarian tumors, intrauterine adhesions, uterine fibroids, pelvic inflammatory disease, and musculoskeletal pain or trauma. However, for a high percentage of patients, true flank pain on physical exam is an indicator of a problem related to the renal system.

Flank pain due to rupture of a renal calyx is an extremely rare occurrence, often resulting from an obstruction. Distinguishing calyceal rupture from infectious etiologies such as pyelonephritis is imperative and involves obtaining a urine culture as well as blood tests, which were done in this patient and found to be within normal limits. In this specific patient, the problem appeared to be obstructive rather than infectious. Examples of obstructive causes include nephrolithiasis, malignancy, pelvic organ prolapse with renal system involvement, and pregnancy. A review of the literature performed in 1995 by Wolff et al. revealed 25 cases of rupture of the urinary tract during pregnancy between the years of 1947 and 1995. 12 patients had ruptures of the collecting system and 13 had ruptures of the renal parenchyma [3]. In 2007, Lo et al. evaluated 17 cases of spontaneous renal rupture during pregnancy, 12 of which occurred in patients with normal kidneys (no underlying explanation for rupture such as infection, abscess, stone, or tumor). All 12 cases of rupture occurred on the right side, likely owing to the fact that hydronephrosis is more common and severe on the right side due to uterine dextroversion. Lo's publication reported the first spontaneous rupture of a normal left kidney during pregnancy [4]. Since the time of Lo's publication, one additional case was published by Matsubara et al. which documented a case of left-sided rupture in a patient with no underlying renal process [5].

Extensive review of the literature in 2013 by Efrimescu et al. was added to the previous body of knowledge and demonstrated 35 total published cases of spontaneous urinary tract rupture during pregnancy, 11 of which occurred on the left side [6]. Although calyceal rupture can occur in kidneys with or without preexisting pathology, a significant proportion of spontaneous upper urinary tract or kidney ruptures during pregnancy are associated with renal calculi [4, 6]. 18 of the 35 cases published by Efrimescu et al. occurred in patients with some form of underlying kidney pathology such as infection, abscess, stones, or tumors, either in the past or at the time of rupture. The case report by Efrimescu et al. documenting the 36th reported case of rupture occurred in a woman with a history of infection [6].

Our patient is the 13th documented case of a left-sided upper urinary tract or kidney rupture in a pregnant woman. Although no definitive stone was appreciated on imaging, this patient did have a history of urolithiasis and was assumed to have urolithiasis at the time of rupture. Renal ultrasound did show small areas of echogenicity which could have been small, nonshadowing stones. As previously detailed, our patient presented with concomitant preterm labor and flank pain and progressed to active labor. She delivered a preterm viable infant vaginally during her hospitalization. It is impossible to determine whether her preterm labor and spontaneous delivery were the cause or the result of her calyceal rupture. However, the suspicion for urolithiasis in this patient is significant because there is a 1.4 to 2.4 times increased risk of premature membrane rupture and preterm labor in pregnant women with symptomatic kidney stones [7]. In a large-scale Canadian study looking at pregnant patients with documented renal calculi, 99% of the calculi occurred in the second or third trimester [8]. Previous studies have shown high rates of preterm labor and preterm delivery in patients with documented or suspected nephrolithiasis. Preterm labor rates range from 5.4% to 20%, and preterm delivery rates range from 2.5% to 13% [9].

Retrospectively, this patient's cause of preterm labor was clearly urologic in nature. However, initially, other causes of preterm labor and abdominal or flank pain must be included in the differential diagnosis. This particular patient had nausea and vomiting at presentation, but metabolic, liver, and biliary function tests were normal, making a gastrointestinal source of symptoms less likely. Additionally, she lacked other gastrointestinal signs and symptoms such as anterior abdominal tenderness and changes in bowel habits. If gastrointestinal or endocrine issues are discovered during an admission workup, it is prudent to involve appropriate disciplines such as internal medicine, gastroenterology, or endocrinology. However, this was not necessary in our particular case. The urology team was appropriately consulted and worked closely with the general obstetricians and maternal-fetal medicine specialists. While pathologic evaluation of the placenta and culture of the amniotic fluid were not performed in this particular case, these are reasonable options in the workup of a possible infectious etiology of preterm labor. A study by Skoll et al. of 127 patients presenting with preterm labor and intact membranes showed that 5.5% of patients had positive amniotic fluid cultures, indicating intrauterine infection. Patients with positive amniotic fluid cultures on admission were found to have a much shorter latency period between time of presentation and delivery compared with patients who had negative fluid cultures, 4.4 days versus 28.6 days, respectively [10]. An intrauterine infection would not explain our patient's calyceal rupture, but it would not have been unreasonable to sample amniotic fluid or send the placenta to pathology in an attempt to locate a source of infection.

While analgesia and conservative therapy are appropriate forms of initial management for patients with symptomatic urolithiasis, patients with pain that persists despite analgesia should receive surgical intervention with percutaneous nephrostomy or stenting [4, 7]. In our patient, failure of conservative management resulted in plans for placement of a ureteral stent. However, before the stent was placed, the patient progressed to active labor and vaginal delivery. Following delivery, our patient's symptoms resolved spontaneously. Our patient's presentation and resolution of symptoms following delivery are similar to previously documented cases of calyceal rupture. Imaging is often unhelpful until rupture has occurred, definitive cause of obstruction is often not found, and once the rupture has occurred, recovery is often uneventful. Conservative management is usually acceptable once rupture has occurred, particularly in cases where the obstructive cause has been relieved. If a documented obstructive cause such as a large renal stone persists following calyceal rupture, more aggressive intervention such as ureteroscopic lithotripsy combined with ureteral stenting is indicated. The natural course of this disease process, however, is resolution of symptoms following rupture as long as no secondary infection develops. Our patient did not receive postdelivery antibiotics, but antibiotics are a reasonable management option once rupture has occurred [11].

Spontaneous rupture of the upper urinary tract is extremely rare during pregnancy, but this case adds to the growing body of medical knowledge related to this topic. Although it is unknown whether early surgical intervention could have prevented this patient's preterm delivery, the changes of pregnancy clearly place an additional stress on the renal parenchyma and collecting system. It is important to recognize the increased risk of delivery associated with both urolithiasis and rupture of the urinary tract, highlighting the need for prompt diagnosis and surgical management when indicated. In this case, it is possible that early surgical intervention with percutaneous nephrostomy tube placement by urology in the antepartum period may have resulted in a more favorable gestational age at time of delivery.

Given the rarity of calyceal ruptures during pregnancy, there have been no publications regarding recurrence rates in patients who experience rupture of the renal system. Further, there are no clear guidelines regarding how to manage this type of patient in the puerperium and how to counsel patients in subsequent pregnancies, highlighting the need for future studies involving long term followup of patients similar to the one described in this case.
